# Medical Miscellanies

**Published:** 1817-12

**Authors:** 


### PART IV.

### MEDICAL MISCELLANIES.

Quicquid agunt Homines.

### *On the Laws of Muscular Motion.* By J. R. Park, M. B. &c.

( Concluded from page 435. )

Such are the grounds on which the conclusion exists, that fatigue is not an exhaustion of nervous energy, but that the moving fibre itself undergoes some change of condition during exertion; that this change, for a time, facilitates and augments the powers of action; then, after a further period, renders its continuance irksome or painful; and finally brings on a state approaching tonic spasm, which partially impedes, or wholly suspends, the power of motion, until rest has relaxed the spasmodic contraction.

But to whatever conclusion we may come, respecting the nature of the changes that occasion these successive stages in the phænomena of motion, their actual occurrence is a matter of observation and experience, admitting of no doubt or dispute: and the laws deduced from them rest entirely upon the facts themselves; their truth and certainty being wholly independent of the accuracy of the explanation, here offered, of the cause that produces them.

The importance of these principles or laws of motion, would be comparatively trifling, if their influence were confined to the class of voluntary organs, which alone we have hitherto considered; but few will be disposed to consider it so, if their operation can be shewn to extend also to those which are involuntary.

Voluntary and involuntary motion do certainly present many striking points of difference; but the question is, whether these be sufficient to constitute them distinct faculties, radically and essentially different in their nature, or only different modifications of the same faculty.

Whytt contended for their identity, and maintained, that involuntary, as well as voluntary organs, derive their power of action immediately from their nerves.

Haller denied their identity, and contended, that nerves, although the chief instruments in producing voluntary motion, have yet nothing to do with that which is involuntary.

Bichât inclines to the same opinion, which, if established, would afford the strongest support to his system; and he has consequently exerted his utmost ingenuity to furnish arguments in its favour, though, after all, he appears afraid to profess it.

In short, there appears no ground for supposing any radical difference between voluntary and involuntary motion, or that nerves, instrumental to the production of the one, are not so to that of the other.

It is true, that contractions may be excited by causes of irritation applied directly to the muscles themselves, as well as to the nerves leading to them. But this circumstance by no means disproves the instrumentality of nerves, since their extreme branches are intimately blended with the structure of the muscle, and inseparable from its substance. This power, moreover, of contracting from direct irritation, is alike possessed by muscles of both classes, and forms therefore no ground of distinction between them.

The brain being equally the immediate organ of volition as well as reflection, the degree in which the motion of any part is subject to the control of the will, as well as the capability each part possesses of exciting mental perceptions, bears a relation to the intimacy of its nervous connection with the sensorium, or the proportion of nerves it derives from the cerebral, and that it receives from the gangliac system.

The internal viscera, the organs of circulation and nutrition, derive their nerves chiefly from the gangliac system, and their actions are consequently automatic, or not directly subject to the influence of the brain.

The blood-vessels shew a state of relaxation, in the retardation of blood, and the swelling of the lower extremities, towards evening, in persons of delicate constitution. As the column of blood bears most on the extremities, it is here that its pressure is first felt, and here that the vessels are soonest overpowered by it. The heart shews its susceptibility of fatigue when fainting occurs, from long standing in the erect posture, without the aid of motion to keep up circulation in the extreme vessels. The stomach is fatigued by long-continued efforts of vomiting; painful sensation attends it, and the powers of digestion are impaired for a time subsequent to this exertion. The exhibition of an active purgative exhausts, in the same way, the power of the intestines, and a degree of constipation usually prevails for a short time after its operation; while the continued use of active purgatives is well known to bring on habitual torpor in the bowels. The urinary bladder, if oppressed by long retention of urine, loses its power of contraction, and strangury ensues. The rectum, in the same way, loses its contractility from long retention and immoderate accumulation of fæces; and obstinate constipation is the result.

The capillary vessels shew the limited extent of their power in a great variety of instances, always by change of function in the organs to which they belong. The cold fit of fever, during which an unusual contraction prevails throughout the capillary system, is followed by a proportionate relaxation and over-distention of those vessels in the hot fit. On the same principle, the more transient constriction of the superficial vessels causing the paleness of fear, is succeeded by an increase of heat and redness, indicating subsequent relaxation, and fulness of vessels in the face and over the surface of the body. The thin watery secretion from the nose, on exposure to severe cold, denoting increased contraction in the secreting vessels, is followed by a dryness and sense of heat in the part, denoting subsequent relaxation and distension of these vessels. Suppressed secretion of urine in hysteria, originating often in the kidnies, and not in the bladder, as proved by the fruitless introduction of the catheter, usually terminates in a secretion remarkably copious. Inordinate or long-continued contraction of the exhalents of the cellular membrane, from exposure to cold and damp, is not unfrequently the cause of dropsy in the lower extremities, denoting loss of tone, and consequent relaxation of these vessels, terminating in serous effusion.

The following instances may serve to illustrate the effects of impaired circulation, or diminished afflux of red blood to the capillary vessels of the moving organs.

Mere inaction, suffering the circulation to languish, frequently produces a kind of nervous tremor, resembling, and often ascribed to the effects of external cold. Extreme cold, which obstructs circulation, and thus impairs the faculty of feeling, also impedes the power of motion; hence the immobility, the tremors, and temporary paralysis, with which the limbs are affected when exposed to its influence. The constriction of the capillaries, produced by certain mental emotions, as fear, and denoted by the paleness and diminished temperature of the surface, is also productive of the same paralytic tremblings and transient loss of power in the limbs. The cold fit of an ague, which also retards circulation, and impedes the afflux of arterial blood to the capillaries, is likewise attended with the same numbness, tremors, and impaired mobility in the muscles. The shrinking of the capillaries attending sea sickness, also causes a diminution of muscular energy, or an aversion to motion, noticed by every one who has experienced it. The weakness and immobility that result from other excessive evacuations, as hæmorrhage, violent purging, &c. equally arise from want of circulation in the capillary vessels, and subside as soon as circulation is restored. In some cases of actual paralysis, where the muscles themselves are the seat of the morbid change, want of circulation still appears to be the immediate cause of loss of power; and accordingly, the means of restoration are all directed to the renewal of active circulation in the muscles. There are also forms of paralysis in which the sensorium, and not the moving organ, appears to be the seat of the morbid change; but here likewise want of circulation is the most probable cause of the diminution of power, and serous effusion only a concomitant circumstance; otherwise, paralysis should always accompany water in the head. In short, every cause that diminishes circulation in the capillary vessels, impairs the vital powers of the part in which this change occurs; and the muscular system, in common with others, is subject to this law.

We shall conclude with an extract from Dr. Johnson's new work on the "INFLUENCE of the ATMOSPHERE," which bears upon the subject discussed by Dr. Park.

"The more intimately we become acquainted with the laws of our own frame, the more we will be convinced that there is no *function* in the body which has not its *alternate* periods of action and repose. This, however, is not the received opinion. One grand division has been made of the functions, into animal or voluntary, and organic or involuntary. The various muscles of locomotion, the muscles by which we masticate our food, &c. belong to the former class—are under the command of the will, and have their periods of repose when we sleep. Those muscles employed in the circulation of the blood, in the digestion of our food, in the secretion and absorption of various fluids, as the bile, &c. belong to the second class—are not under the will, and are *supposed* by physiologists to go on uninterruptedly, both while asleep and awake. The intellectual system has also its alternations of exertion and rest; for when, in sleep, the senses, as hearing, feeling, &c. cease to transmit impressions to the brain, the mind has no longer materials to act on, and immediately becomes unconscious of its own existence.*

"This law of the intermission of action has been applied by Bichât to the theory of sleep. "General sleep," says he, "is the assemblage of particular sleeps. It is derived from that law of the animal life which causes in its functions a constant succession of periods of activity, and times of intermission; *a law which pointedly distinguishes itself from the organic life.*" Now, I do not think that this *point of distinction* is so fairly made out as the celebrated Frenchman supposes. Let us take one of the viscera belonging to the organic life—say the heart, which is the *punctum saliens* in the embryo, and the *ultimum moriens* in old age. The time occupied in each systole or contraction of the ventricle is about the third of a second; and that required for the diastole or relaxation, two thirds of a second. Here then the heart, the most active muscle belonging to the organic life, *rests* sixteen hours in every twenty-four.

"It may be objected to this, that in the diastole of the ventricle a motion of self expansion is produced; and consequently, that the walls of the heart themselves are not then at rest. But this principle of self expansion, if it do exist, must be produced by the elastic matter interspersed among the muscular fibres; for it will hardly be contended, that muscular action, which must be muscular contraction, can have the effect of dilating the heart. In every point of view then this central and laborious organ of the circulation has its "*periods of activity, and times of intermission,*" just as regularly, if not more so, than any voluntary muscle in the human frame.

"The same may be said of the stomach, intestines, liver, lungs, and all the organs of secretion, excretion, and absorption. They are governed by the same general laws of *action and repose*, that regulate the voluntary muscles, the organs of sense, and the intellectual functions. Nor is this a useless or merely curious discussion. It teaches us an instructive lesson, in shewing us, that the *organic functions* require *their* periods of sleep as well as our wearied voluntary muscles; a consideration which seldom seems to have entered the thoughts of those who goad them on to almost incessant exertions, as though they were inanimate machines, endowed with the power of *perpetual motion.*" P. 149.

---

### *Reply to Dr. Von Embden.*

One of the Medico-Chirurgical Reviewers has seen, with some surprise, the anathema which Dr. Von Embden has thought fit to level against him in the last Number of the *London Medical and Physical Journal.* The critique is characterized as the effusion of

---

* This statement refers, of course, to natural and sound sleep. Dreams will be noticed hereafter.

a "Gall-dipped pen of an *illiberal* individual—an effusion which none but the *hapless reviewer* could write." The Medico-Chirurgical writer may be a "hapless reviewer," but he is *not a literary pedlar*, who hawks about his *manufactured* articles of foreign growth through the English markets.* His writings are not the offsprings of a *mercenary* spirit. His pen may have been dipped in gall; but it was never prostituted at the shrine of *usury;* nor will it ever fear to unmask the *Israelite*, when he assumes the garb of the medical philosopher.

And how has Dr. Von Embden excused himself for the matchless absurdities which were pointed out in the critique?—Because forsooth—"no candid reader would hesitate a moment to lay them to the charge of the *composer and reviser.*" Thus the learned Editor of the Continental Repertory has nothing to do with the *composition or revisal* of a work which he modestly presents to his friend, John Bull, as carefully translated and selected from the Medical Literature of the Continent!

An artful appeal is made to the English reader through the medium of an *Editorial Certificate*, that the Philippic in the Medical and Physical Journal is "*printed exactly as they received it,*" thereby insinuating, that it is printed exactly as Dr. Von Embden *wrote it*, and, consequently, that the accusation of the Medico-Chirurgical writer, respecting Dr. Von Embden's incompetency in the English language for the Editorship of an English work, was gross, illiberal—nay false. But the Medico-Chirurgical writer is not such a simpleton to be baffled by this manœuvre. He fearlessly asserts, and he appeals to any or every man of literature for the truth of his assertion, that the style and composition of the Continental Medical and Physical Repertory, and those of the letter of the English Journal, are so totally and distinctly opposite, that they would afford evidence on oath, if required, in a court of law, that they were the productions of different writers. Would the veriest tyro in the profession believe that the man, who, in describing the symptoms of a fever, *repeatedly* substituted

* A few months ago a medical gentleman just returned from the Continent called on the writer of this paper, and among other conversation, enquired how the Hamburgh *manufactures* went off in the medical world here? He answered, that he did not understand the purport of the question; when the gentleman pointed out "numerous communications in almost all the medical periodical publications in England and Scotland," [vide *last Medical Journal*] observing, that they occasioned no little merriment in certain parts of the Continent at the *credulity* of John Bull! A few days after this, the writer received a letter from town, informing him, that a demand had been made on the *Medico-Chirurgical Journal*, by a Hamburgh Manufacturer, of *two guineas for every fourteen pages* of Articles from that depôt!—The answer which was made to this demand may be easily guessed at by a glance at the pages of the Medico-Chirurgical Journal since that period. A number of other anecdotes came forth at this time, which, when the plot thickens a little more, will be laid before John Bull, in due form.

*frost* for cold,* was capable of writing the letter in the Medical and Physical Journal? No indeed. The *Repertory* bears such intrinsic marks of a half-English foreigner—such, for instance, as *a half naturalized Jew,* and the *letter* those of the GRINDER, that the whole manœuvre recoils on the heads of the inventors, as a base and disingenuous subterfuge, unworthy of a man of literature—disgraceful to science and the profession! Lastly, Dr. Von Embden and his organ, the GRINDER, affect to hold the criticisms of the Medico-Chirurgical writer in the most sovereign contempt; but their long, laboured, and virulent fulmination, in which Greek, Latin and English verses are brandished as incantations to frighten him from the field, discloses a sentiment very different indeed from that of *contempt!* How far the contemned critique shall—

"Teach them to mourn their 'Journal'—or to mend,"

may probably appear in the sequel.

*November* 15, 1817. MEDICO-CHIRURGICUS.

* Two instances of this occur in page 94. "Without warning, the patient is taken suddenly with *frost,* whilst inwardly, &c." And again, "The symptoms are the same as before, only that they are more violent, and without any *frost.*"

---

Dr. MERRIMAN and Dr. LEY will begin another Course of Lectures on Midwifery and the Diseases of Women and Children, early in December, at the Middlesex Hospital.

Dr. GORDON has just published his Outlines of Lectures on Human Physiology.

DEATHS.—At Ellesmere, Mr. James, Surgeon.—At Buntingford, Robert Wood, M. D.

